# Projected Influences of Changes in Weather Severity on Autumn-Winter Distributions of Dabbling Ducks in the Mississippi and Atlantic Flyways during the Twenty-First Century

**DOI:** 10.1371/journal.pone.0167506

**Published:** 2016-12-13

**Authors:** Michael Notaro, Michael Schummer, Yafang Zhong, Stephen Vavrus, Lena Van Den Elsen, John Coluccy, Christopher Hoving

**Affiliations:** 1 Nelson Institute Center for Climatic Research, University of Wisconsin-Madison, Madison, Wisconsin, United States of America; 2 Department of Biological Sciences, State University of New York at Oswego, Oswego, New York, United States of America; 3 Space Science and Engineering Center, University of Wisconsin-Madison, Madison, Wisconsin, United States of America; 4 Long Point Waterfowl/Bird Studies Canada, Port Rowan, Ontario, Canada; 5 Ducks Unlimited, Ann Arbor, Michigan, United States of America; 6 Michigan Department of Natural Resources–Wildlife Division, Lansing, Michigan, United States of America; Linnaeus University, SWEDEN

## Abstract

Projected changes in the relative abundance and timing of autumn-winter migration are assessed for seven dabbling duck species across the Mississippi and Atlantic Flyways for the mid- and late 21^st^ century. Species-specific observed relationships are established between cumulative weather severity in autumn-winter and duck population rate of change. Dynamically downscaled projections of weather severity are developed using a high-resolution regional climate model, interactively coupled to a one-dimensional lake model to represent the Great Lakes and associated lake-effect snowfall. Based on the observed relationships and downscaled climate projections of rising air temperatures and reduced snow cover, delayed autumn-winter migration is expected for all species, with the least delays for the Northern Pintail and the greatest delays for the Mallard. Indeed, the Mallard, the most common and widespread duck in North America, may overwinter in the Great Lakes region by the late 21^st^ century. This highlights the importance of protecting and restoring wetlands across the mid-latitudes of North America, including the Great Lakes Basin, because dabbling ducks are likely to spend more time there, which would impact existing wetlands through increased foraging pressure. Furthermore, inconsistency in the timing and intensity of the traditional autumn-winter migration of dabbling ducks in the Mississippi and Atlantic Flyways could have social and economic consequences to communities to the south, where hunting and birdwatching would be affected.

## Introduction

Waterfowl and their habitats are ecologically, economically, and socially valuable, providing food, income, recreation, and ecosystem stability [1]. It has been estimated that 1.8 million waterfowl hunters in the United States participate during nearly 30 million recreational days per year, with the economic activity leading to $2.3 billion in spending and over 21,000 jobs [2]. The total economic impact of waterfowl hunting in Mississippi alone was estimated at $27.4 million [3]. An estimated 47 million birdwatchers in the United States, over the age of 16, spent approximately $41 billion in total trip and equipment expenditures in 2011, with peak participation in the South and birding expenditures supporting 666,000 jobs [4]. The most watched type of bird for traveling birders is waterfowl [4].

Because waterfowl are relatively large-bodied migratory birds that number in the millions, they have considerable energy needs during migration and winter [5]. Redistribution of these birds under climate change would result in substantial changes in their latitudinal foraging pressure in aquatic and agricultural habitats [6]. Despite its importance to managers and conservation planners, there has been insufficient exploration of the meteorological factors influencing the movement and distribution of waterfowl during the autumn-winter non-breeding period, with impacts on waterfowl harvest, habitat use, and survival [5,7–13]. While the impact of climate change on birds has received considerable attention, these studies have primarily focused on the breeding season, and few studies have investigated changes to the distributions of waterfowl during autumn-winter [13–17].

The autumn migration of dabbling ducks is thought to be controlled by changes in photoperiod; habitat suitability and management; food accessibility; weather severity, including effects from both temperature (thermoregulatory cost) and snow cover; feeding ecology and competition [7,12,17–32]. The cost of migration is high [33–34]. However, when temperature is below a critical threshold, elevated thermoregulatory demands cause it to become more energetically costly for Northern Hemispheric waterfowl to persist at higher latitudes than to migrate southward to warmer environments, according to energy conservation theory [5,35–37]. Furthermore, the presence of snow and ice cover can restrict wetland food availability and foraging capacity, thereby increasing competition and time in flight and reducing nutrient acquisition among wetland-obligate waterfowl [38–40]. [41], for example, demonstrated that winter temperature and snow cover are key regulators of waterfowl abundance in the Mississippi Alluvial Valley, which is the primary region for wintering Mallards (*Anas platyrhynchos*) [7,42]. Waterfowl, such as Mallards, typically migrate farther south in response to cold conditions in their northern wintering areas [7,43–45], with movements often triggered by cold spells [19,30, 40,46–48] or food depletion [30,49–50].

Anthropogenic climate change, particularly rising air temperatures, longer growing seasons, and diminished snow accumulation at many locations [51], has already induced noteworthy phenological shifts in the timing of migration and breeding by waterfowl and other birds, thereby impacting population distributions, and poleward shifts in range margins [13,45, 52–70]; however, attributing these range shifts to specific drivers is often not straightforward [31–32]. For example, the abundance of American Black Duck (*Anas rubripes*), a short-distance migrant, has shown signs of delayed migration and wintering at more northern areas [18,67,71–74]. A continued poleward shift in the distribution of wintering waterfowl will increase foraging pressure and, thus, habitat needs across mid-high latitudes, including central North America, where only 10–15% of historical wetlands remain [62,75–78].

Recent studies by [6,12,21,23] have focused on the relationship between changes in the relative abundance of dabbling ducks and meteorological variables across mid-latitude North America. Based on waterfowl survey data from conservation areas, [12] developed a cumulative weather severity index (WSI) to explain changes in the relative abundance of Mallards during autumn-winter migration at their staging areas in Missouri, within the Mississippi Flyway. Their model represented the current and cumulative effects of air temperature on energy expenditure and snow cover and wetland icing on food availability by considering four variables: (1) the mean daily temperature, (2) the number of consecutive days with mean air temperature at or below freezing, (3) snow depth, and (4) the number of consecutive days with at least 2.54 cm of snowpack. The WSI is a valuable tool for resource managers and researchers to identify the days when Mallards are likely to begin leaving a location for more southern latitudes [21]. [23] later expanded the analysis to the meteorological and photoperiod regulators of the rate of change in the relative autumn-winter abundance of multiple dabbling duck species using aerial and ground-based survey data across the Mississippi and Atlantic Flyways for 25 locations in the United States and Canada. The statistical models that explained the greatest total variance included air temperature, snow depth, and latitude for all analyzed duck species, except for the Blue-Winged Teal (*Anas discors*), which is a long-distance migrant that largely responds to photoperiod. In general, the primary migration cue for long-distance migrants (i.e., neotropical migrants) wintering in stable habitats is photoperiod, with decreasing day length synchronizing their circadian and circannual rhythms, while weather cues have a greater impact on short-distance migrants that winter in unstable habitats [23,79–80]. Most dabbling ducks that breed in North America winter at southern locations on the continent, where they are subject to variable weather conditions during the non-breeding period [22].

[81] investigated future changes in the relative abundance and autumn-winter migratory timing of Mallards across central-eastern North America based on the WSI established by [12]. Projected changes in air temperature and snow depth by the mid- and late 21^st^ century were based on statistically downscaled climate projections from nine global climate models (GCMs) within the Coupled Model Intercomparison Project Phase Three (CMIP3) and the application of an operational snow accumulation and ablation model. December-January WSI was projected to decline substantially during this century, leading to increased likelihood of delays in the timing and intensity of autumn-winter waterfowl migrations. Besides being restricted to only one dabbling duck species, the previous study was limited because the statistical downscaling approach used to derive snowfall projections did not consider projected changes in water temperature, ice cover, and evaporation for the Great Lakes and thus the earlier projections poorly represented future lake-effect snowfall dynamics [81]. This is particularly concerning, given the pronounced trends of declining ice cover [82] and increasing lake evaporation [83] and lake-effect snowfall [84,85] that have already been observed. To address this limitation, [86,87] produced dynamically downscaled climate projections for the Great Lakes Basin and broader region using a high-resolution regional climate model (RCM), interactively coupled to a one-dimensional lake model to represent changing water temperature and ice cover on the Great Lakes. They downscaled two of the Coupled Model Intercomparison Project Phase Five (CMIP5) GCMs. Here, we expanded the number of downscaled GCMs to six in order to better capture uncertainty of regional climate change projections.

The objective of the current study is to investigate the likely impacts of dynamically downscaled weather severity projections for the mid- and late 21^st^ century on the relative abundance and autumn-winter migratory behavior of seven common dabbling duck species across the Mississippi and Atlantic Flyways of North America. While the current study focuses on dabbling ducks as an example of the application of downscaled weather severity projections, the general approach can be more broadly applied to other wildlife investigations.

## Data and Methods

### Species-specific weather severity indices

The following summarizes the statistical models developed by [23] for the relative rate of change of seven focal dabbling duck species as a function of current and cumulative weather severity. The onset of negative population rates implies southward migration. [23] applied an information-theoretic approach for model selection [88] based on Akaike’s Information Criterion (AIC), which measures the quality of statistical models while penalizing based on the number of predictors. All candidate models within 2.0 ΔAIC units of the top-ranked models were believed to exhibit biological significance, such that modeling averaging was applied to estimate parameters and the 85% confidence intervals for the top models [89]. All calculations are performed during September through March, focusing on the autumn-winter migration.

For the American Black Duck, principal component (PC) analysis of the individual elements of weather severity yields the following index for PC1:

PC1=0.965×TEMP+0.171×TEMPDAY+0.031×SNOW+0.197×SNOWDAY (1)

where TEMP is the daily mean air temperature (°C) multiplied by -1, TEMPDAY is the number of consecutive days with mean air temperature less than or equal to 0°C, SNOW is the daily snow depth (cm) multiplied by 0.394, and SNOWDAY is the number of consecutive days with at least 2.54 cm of snow on the ground. The PC analysis aims to produce an index that explains the maximum variance among TEMP, TEMPDAY, SNOW, and SNOWDAY, which are seasonally correlated [12,90]. The empirical coefficients in Eq (1) represent the eigenvectors of the predictor variables that compose the first PC. Based on the PC1 index and latitude (degrees north), the following quadratic equation estimates the relative rate of change in American Black Duck abundance between two dates at a surveyed location:

$${Rate}_{ABD}=-0.012\times {PC1}^{2}–0.005\times {LAT}^{2}–0.134\times PC1+0.442\times LAT–7.900.$$ (2)

Likewise, for Mallards, the PC1 index and resulting rate were as follows:

PC1=0.932×TEMP+0.235×TEMPDAY+0.051×SNOW+0.270×SNOWDAY (3)

$${Rate}_{M}=-0.008\times {PC}^{2}-0.002\times {LAT}^{2}+0.008\times PC1\times LAT–0.410\times PC1+0.193\times LAT–3.637.$$ (4)

Sample data for the calculation of Mallard population rate is presented in S1 Table to demonstrate the methodology. For the American Wigeon (*Anas americana*), the index, WSIMEAN, is introduced and defined as:

WSIMEAN=TEMPMEAN+TEMPDAY+SNOW+SNOWDAY, (5)

where TEMPMEAN is the mean air temperature during the last seven days multiplied by -1. The relative rate of change of population for the American Wigeon is estimated by:

$${Rate}_{AW}=0.002\times {WSIMEAN}^{2}+0.043\times {LAT}^{2}–0.005\times WSIMEAN\times LAT–0.049\times WSIMEAN–3.599\times LAT+72.218.$$ (6)

The rates for Gadwall (*Anas strepera*, Rate_G_), Green-Winged Teal (*Anas crecca*, Rate_GWT_), and Northern Shoveler (*Anas clypeata*) are as follows:

$${Rate}_{G}=0.002\times {WSIMEAN}^{2}+0.019\times {LAT}^{2}-0.011\times WSIMEAN\times LAT+0.175\times WSIMEAN–1.474\times LAT+26.575$$ (7)

$${Rate}_{GWT}=-0.002\times {WSIMEAN}^{2}+0.007\times {LAT}^{2}–0.017\times WSIMEAN\times LAT+0.396\times WSIMEAN–0.810\times LAT+19.050$$ (8)

$${Rate}_{NS}=-0.001\times {WSIMEAN}^{2}+0.022\times {LAT}^{2}–0.001\times WSIMEAN\times LAT–0.188\times WSIMEAN–1.805\times LAT+34.087.$$ (9)

For Northern Pintain (*Anas acuta*), the WSI index is computed as follows,

WSI=TEMP+TEMPDAY+SNOW+SNOWDAY (10)

in which the maximum value of WSI over the last seven days is selected. Using this WSI index, the relative rate of change of population for the Northern Pintail is estimated by:

$${Rate}_{NP}=-0.001\times {WSI}^{2}+0.006\times {LAT}^{2}–0.106\times WSI\times LAT+0.693\times WSI–0.618\times LAT+13.311.$$ (11)

Among the numerous candidate models adapted from [12], [23] selected WSIMEAN to be retained in the best models for the American Wigeon, Gadwall, Green-Winged Teal, and Northern Shoveler only and selected WSI to be retained in the best model for the Northern Pintail only.

Given the quadratic fit of most of the aforementioned population rate formulas, biologically unrealistic values can be generated for PC1 and WSIMEAN by the aforementioned models when WSI is calculated for extremely mild or severe values not within the range of the collected data sample. As a result, positive population rates are assumed in the study region for the American Black Duck when PC1 falls below the threshold of -19 to -15 (depending on latitude) or for the Mallard when PC1 falls below the threshold of -23 to -11, indicative of mild, snow free conditions. Furthermore, negative population rates are assumed for the American Wigeon and Gadwall when WSIMEAN exceeds the thresholds of 85 to 150 and 80 to 185, respectively, indicative of cold, snow-covered conditions.

### Dynamical downscaling

**Fig 1:**
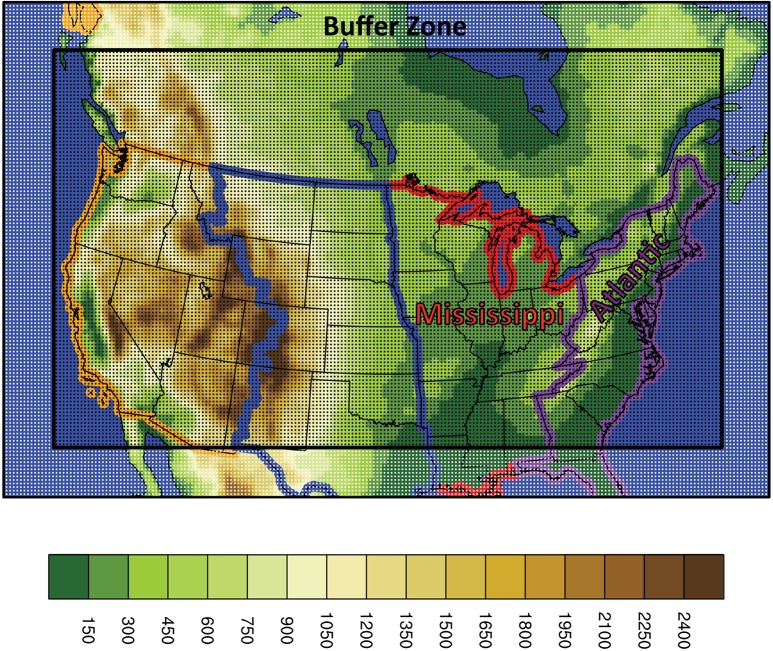
Model domain with elevation (shading, meters). The thick black rectangle indicates the buffer zone. Dots indicate the 25-km grid spacing. The orange, blue, red, and purple polygons identify the Pacific, Central, Mississippi, and Atlantic Flyways, respectively, based on a shapefile obtained from Ducks Unlimited.

The output from six CMIP5 GCMs is dynamically downscaled using the Abdus Salam International Centre for Theoretical Physics (ICTP) Regional Climate Model version four (RegCM4) [91], interactively coupled to a one-dimensional, energy-balance lake model [92] and lake ice sub-model [93–94] to represent the Laurentian Great Lakes. The GCMs include the Centre National de Recherches Meteorologiques Coupled Global Climate Model Version Five (CNRM-CM5), the Model for Interdisciplinary Research on Climate Version Five (MIROC5), the Institut Pierre Simon Laplace Coupled Model Version Five-Medium Resolution (IPSL-CM5-MR), the Meteorological Research Institute Coupled Global Climate Model Version Three (MRI-CGCM3), the Centre for Australian Weather and Climate Research, Australia GCM (ACCESS1-0), and the National Oceanic and Atmospheric Administration Geophysical Fluid Dynamics Laboratory model (GFDL-ESM2M). The domain, consisting of 217 by 141 grid cells, extends across most of the contiguous United States and southern Canada (Fig 1). The simulations apply 25-km grid spacing and 28 vertical sigma levels. Lateral boundary conditions from the GCMs are provided through a linear relaxation scheme to a 15 gridcell buffer zone, which surrounds the inner domain. Analyses for the late 20^th^, mid-21^st^, and late 21^st^ centuries are limited here to 1980–1999, 2040–2059, and 2080–2099, according to the representative concentration pathway 8.5 (RCP8.5) [95], which is a high-end emission scenario for greenhouse gases. Further information on the dynamical downscaling and model performance is presented by [86–87,96–99]. The locations of the Mississippi and Atlantic Flyways within the United States are identified in Fig 1. Subsequent area-average calculations for the study region cover 30–50°N, 97–67°W, extending northward into the breeding zones across southern Ontario, Quebec, and Manitoba.

### Debiasing methodology

Prior to calculating species-specific weather severity indices, RegCM4-simulated output of daily 2-meter air temperature and snow depth is debiased against observational data, both in terms of the daily mean and interannual standard deviation for each day. The source of observed daily mean air temperature for 1984–2013 is the 1-km, gridded Daily Surface Weather and Climatological Summaries (Daymet) product [100–101]. For the purpose of debiasing daily snow depth, a gridded product is created using data from 7,360 meteorological stations within the Global Historical Climate Network (GHCN) [102] across the area of 26–54°N, 101–63°W; this station list is reduced from the original 12,911 stations in that region by requiring at least 16.7% daily data availability. For a given day, debiasing is performed by subtracting the simulated climatological mean for that day, multiplying by the ratio of the observed interannual standard deviation to the simulated interannual standard deviation for that day, and then adding the observed climatological mean for that day. [103] explored multiple debiasing techniques, including linear bias correction of the mean and standard deviation as applied here, and concluded that the relative performance of each method varies by region and season, such that no universally superior method could be identified. Subsequent analyses and plots apply the debiased temperature and snow data.

## Results

### Projected climate change

**Fig 2:**
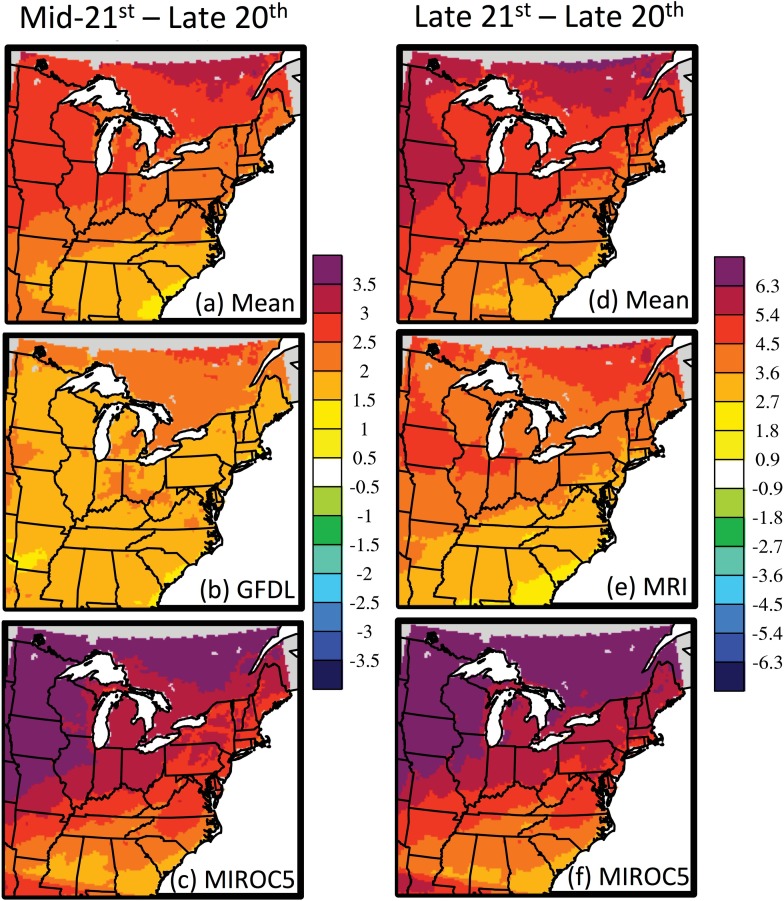
**Projected change in 2-m air temperature (°C) for autumn-winter (September through February) by the (a-c) mid-21**^**st**^ **and (d-f) late 21**^**st**^ **century, compared to the late 20**^**th**^ **century.** Results are shown for the (a,d) six-model mean, (b,e) the model with the least warming, and (c,f) the model with the greatest warming.

According to the debiased dynamical downscaling, the Mississippi and Atlantic Flyways are projected to warm in autumn-winter (September through February) by 2.4°C by the mid-21^st^ century, ranging from +1.9°C for GFDL to +3.1°C for MIROC5, compared to the late 20^th^ century (Fig 2A–2C). The mean projected warming by the late 21^st^ century is 4.8°C, ranging from 3.9°C for MRI to 5.7°C for MIROC5 (Fig 2D–2F). The uncertainty in projected warming, represented by the spread among models, increases substantially later in the century. The projected warming is approximately 10% greater in the debiased data than the original RCM data, due to the model’s modest underestimation of the interannual variability in temperature. For all six models and both time periods, the peak projected warming occurs over southern Ontario, Quebec, and Manitoba and the Upper Midwest United States, representing the northern and western portions of the study region. There are also local minima in warming in close proximity to the Great Lakes and Atlantic Coast, due to the buffering effect of these large water bodies and their substantial heat capacity.

**Fig 3:**
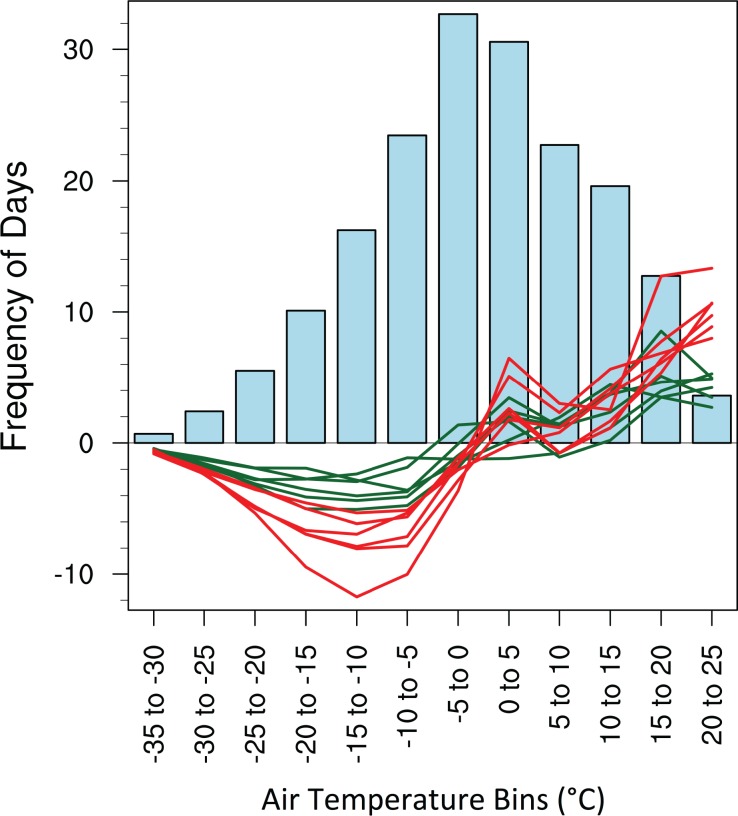
Projected change in the frequency of days during autumn-winter (September-February) with daily mean air temperatures lying within specified bins between -35°C to -30°C and 20°C to 25°C across the Great Lakes region (40–50°N, 95–70°W). The frequency for the late 20^th^ century is shown in blue bars. Projected changes in frequency by the mid- and late 21^st^ century, compared to the late 20^th^ century, are shown with green and red lines, respectively, with one line for each of the six downscaled models.

The frequency of extremely cold episodes across the Great Lakes region (40–50°N, 95–70°W) during autumn-winter (September through February) is expected to decrease substantially during the 21^st^ century, based on an analysis of the frequency of days within specific mean air temperature bins, between -35°C to -30°C and 20°C to 25°C (Fig 3). During the late 20^th^ century, 35% of days exhibit a mean temperature within 5°C of the freezing point, with -5°C to 0°C and 0°C to 5°C representing the most active bins. The frequency of days below freezing is expected to decrease and the frequency of days above freezing is expected to increase during the 21^st^ century within the Great Lakes region as the probability density function of daily air temperatures experiences a pronounced shift towards higher temperatures. As a result of this warming, the projected increase in autumn-winter precipitation across the Great Lakes region among all six models is characterized by greater rainfall and reduced snowfall. Specifically, when averaged across the six models, the largest projected decline in frequency is -3.6 days by the mid-21^st^ century and -7.7 days by the late 21^st^ century for the -15°C to -10°C bin, and the largest projected increase in frequency is +4.3 days by the mid-21^st^ century and +10.2 days by the late 21^st^ century for the 20°C to 25°C bin.

In response to the aforementioned warming in autumn-winter, ice cover on the Great Lakes is projected to experience pronounced reductions during the 21^st^ century, especially in February-March, with declines during the two-month period ranging from -31% in GFDL to -50% in MIROC5 by the late 21^st^ century (not shown). Declines of this magnitude would mean that the Great Lakes are projected to become mostly open water during winter by the late 21^st^ century, with a dramatically shortened ice season. As described by [86], future changes in lake-effect snowfall are uncertain; declining lake ice cover would support more evaporation and thus more lake-effect snowfall, but the reduced frequency of strong cold air outbreaks out of Canada would trigger fewer lake-effect snow events. [86,96] introduced an objective methodology for identifying heavy lake-effect snowstorm days in RCM output, based on proximity to lakeshore, wind direction, lake ice cover, local snowfall amount, and enhancement of snowfall near the lakeshore. Based on the application of these criteria, the frequency of heavy lake-effect snowstorms within the Great Lakes Basin is projected to decline by -1% in GFDL to -20% in ACCESS by the mid-21^st^ century and by -17% in GFDL to -45% in ACCESS by the late 21^st^ century.

**Fig 4:**
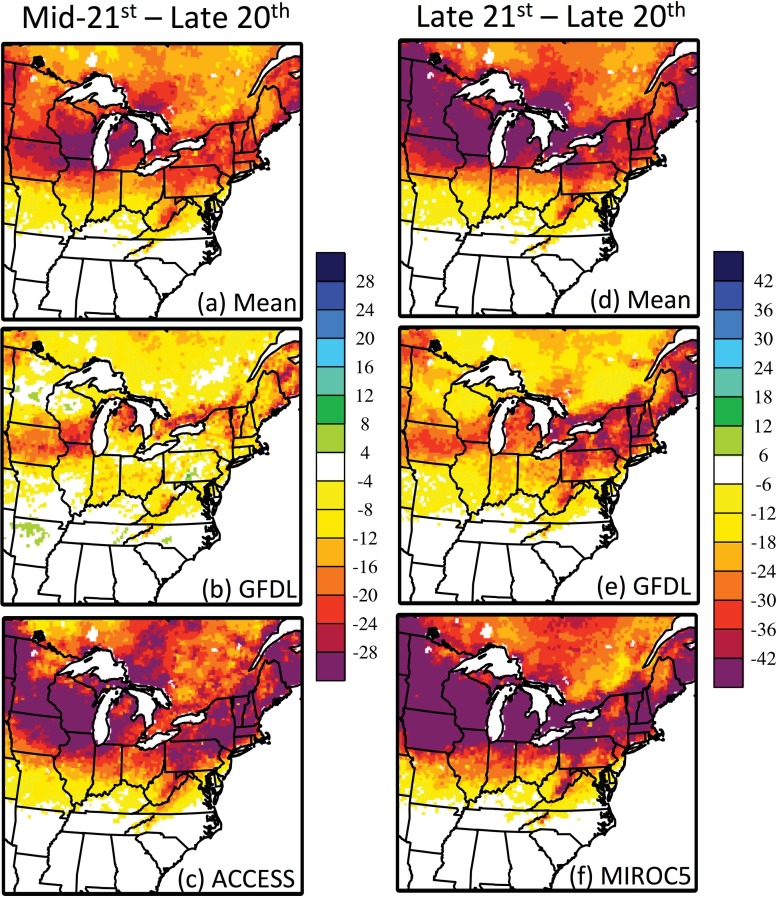
**Projected change in the mean number of days during autumn-winter (September through February) with at least 2.54 cm of snow on the ground by the (a-c) mid-21**^**st**^ **and (d-f) late 21**^**st**^ **century, compared to the late 20**^**th**^ **century.** Results are shown for the (a,d) six-model mean, (b,e) the model with the least loss of snowpack, and (c,f) the model with the greatest loss of snowpack.

Due to reduced snowfall and accelerated snowmelt, the mean number of days with at least 2.54 cm (1 inch) of snow on the ground is projected to decline across the Mississippi and Atlantic Flyways (Fig 4). Reductions of -8.5 days (-27%, compared to 31.1 days in the late 20^th^ century) are simulated by the mid-21^st^ century, ranging from -4.3 days in GFDL to -10.9 days in ACCESS, and of -15.0 days (-48%) are simulated by the late 21^st^ century, ranging from -10.4 days in GFDL to -17.8 days in MIROC5. Within the study region, the zone of most pronounced reductions in the duration of snowpack is identified around 44–45°N, across the Great Lakes region, partly associated with the projected decline in lake-effect snowfall.

### Projected responses of dabbling ducks

**Fig 5:**
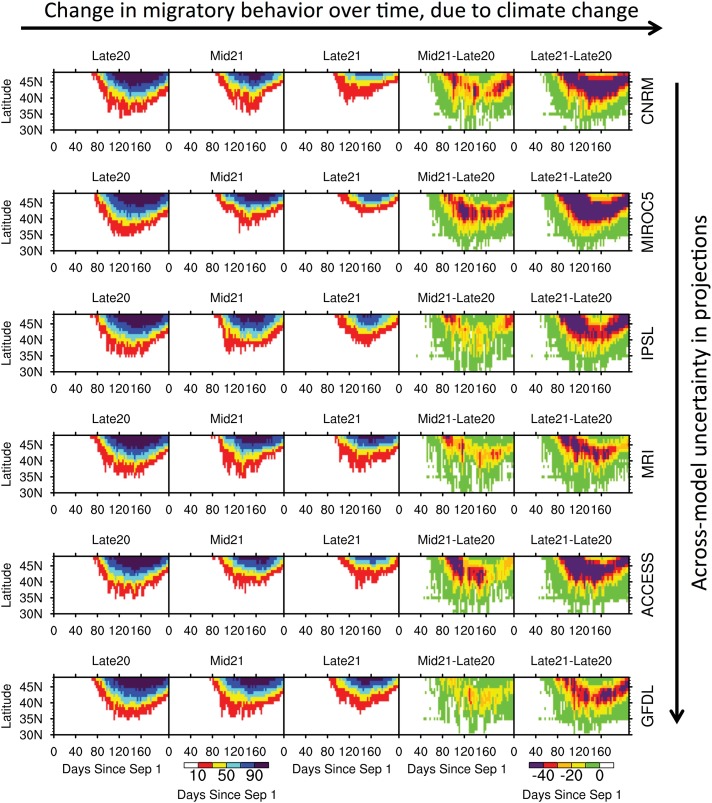
Probability of a negative population rate of the Mallard across the Mississippi and Atlantic Flyway, within latitudinal bands from 30°N to 50°N, for the late 20^th^, mid-21^st^, and late 21^st^ centuries (first three columns). The probabilities are shown for each day from 1 September through 31 March, with the x-axis labeled as days since 1 September. Projected changes in these probabilities are shown for the mid-21^st^ and late 21^st^ century, compared to the late 20^th^ century (fourth and fifth columns). Results are shown for CNRM, MIROC5, IPSL, MRI, ACCESS, and GFDL, from top to bottom row.

**Fig 6:**
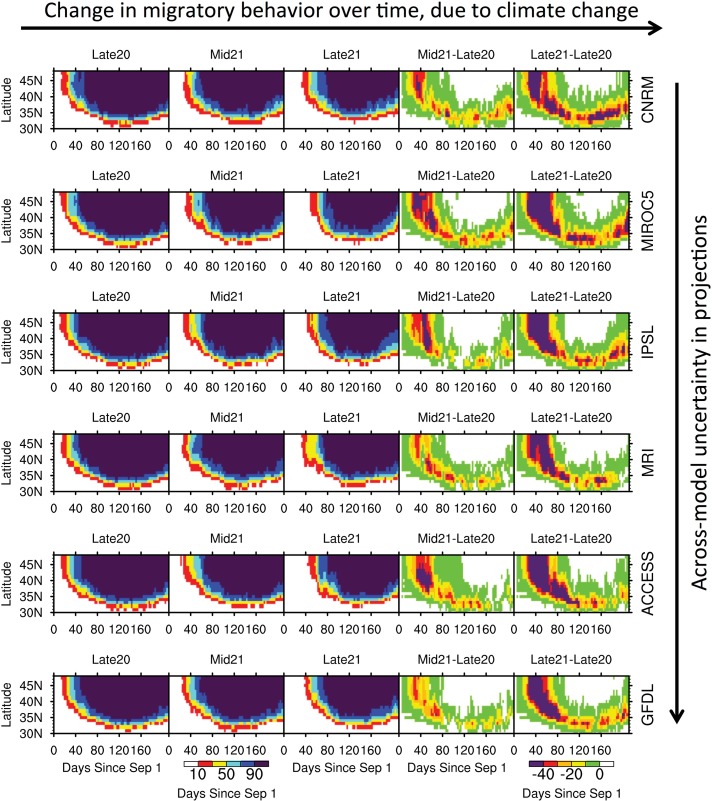
Same as Fig 5, but for the Northern Shoveler.

The mean migration date from the Great Lakes region for seven dabbling ducks species is estimated according to the aforementioned weather severity equations across the Great Lakes zone of 40–50°N (Table 1). Mean migration date is defined as the initial date during autumn-winter in which there is at least a 50% chance (computed across years) of achieving a negative population rate due to low temperatures and snowpack. Among the seven species, the American Black Duck and Mallard are the latest migrants, with mean migration dates out of the Great Lakes region of 10 December and 9 December, respectively, during the late 20^th^ century (Figs 5 and S1). In contrast, the Northern Shoveler is the earliest migrant for the same region, with a mean migration date of 2 October during the late 20^th^ century (Fig 6).

**Table 1:** Summary for seven analyzed dabbling duck species.

Duck Species	(a) Mean Migration Date from Great Lakes region: Late 20^th^ Century	(b) Mean Migration Date: Mid-21^st^ Century, Earliest and Latest Model	(c) Mean Migration Date: Late 21^st^ Century, Earliest and Latest Model	(d) Change: Mid-21^st^ Century Minus Late 20^th^ Century	(e) Change: Late 21^st^ Century Minus Late 20^th^ Century
American Black Duck	10 Dec (Late Migrant)	GFDL: 18 Dec MIROC5: 8 Jan	MRI: 7 Jan MIROC5: Never	+19 days	+33 days, if ever
American Wigeon	16 Oct	CNRM: 26 Oct MIROC5: 8 Nov	CNRM: 4 Nov ACCESS: 17 Nov	+15 days	+27 days
Gadwall	5 Nov	GFDL: 15 Nov MIROC5: 20 Nov	GFDL: 23 Nov ACCESS: 8 Dec	+13 days	+24 days
Green Winged Teal	15 Oct	CNRM: 25 Oct MIROC5: 7 Nov	CNRM: 3 Nov MIROC5: 15 Nov	+16 days	+25 days
Mallard	9 Dec	GFDL: 18 Dec MIROC5: 5 Jan	GFDL: 5 Jan MIROC5: Never	+19 days	+40 days, if ever
Northern Pintail	4 Nov	GFDL: 14 Nov MIROC5: 20 Nov	GFDL: 21 Nov MIROC5: 7 Dec	+12 days	+23 days
Northern Shoveler	2 Oct (Early Migrant)	GFDL: 11 Oct IPSL: 24 Oct	CNRM: 23 Oct MIROC5: 7 Nov	+15 days	+29 days

For each of seven duck species, the following information is provided for the Great Lakes zone of 40–50°N: (a) mean migration date from the Great Lakes region during the late 20^th^ century, (b) earliest and latest mean migration dates during the mid-21^st^ century among the six models, (c) earliest and latest mean migration dates during the late 21^st^ century among the six models, (d) change in the mean migration date by the mid-21^st^ century, compared to the late 20^th^ century, and (e) change in the mean migration date by the late 21^st^ century, compared to the late 20^th^ century.

Both the timing and the distance traveled (implied if species do not have to leave northern latitudes due to weather severity) during migration are projected to shift for each of the dabbling duck species. Projected changes in the probability (among 20 years) of negative population rates across the Mississippi and Atlantic Flyways, and thus the tendency for autumn-winter migration, are presented in Figs 5 and 6, for the Mallard (late migrant) and Northern Shoveler (early migrant) as contrasting examples, and S1–S5 Figs, for the remaining species, through zonal averages (by latitude from 30°N to 50°N). The probabilities are shown for each day from 1 September through 31 March (e.g. 1 Jan is day 122), with the x-axis labeled as days since 1 September. In terms of the two rightmost columns in Figs 5 and 6, which display projected changes in the probability of a negative population rate, a shift to the right of the colored shading indicates a delayed phenological response in migration, while a shift upward indicates the tendency for more southerly populations to overwinter and not migrate.

In general, the greatest (least) projected delays in migration are expected for modern-day late (early) migrant species. The Northern Pintail is projected to experience the least delay in migration out of the Great Lakes region (40–50°N), on the order of +12 days and +23 days by the mid- and late 21^st^ century, when averaged across the six models (Table 1, S5 Fig). Its mean migration date is 4 November in the late 20^th^ century, ranges from 14 November in GFDL to 20 November in MIROC5 for the mid-21^st^ century, and ranges from 21 November in GFDL and 7 December in MIROC5 for the late 21^st^ century. The greatest delay in migration out of the Great Lakes region is expected for the Mallard, on the order of +19 days by the mid-21^st^ century and +40 days, or the possibility of overwintering, by the late 21^st^ century (Table 1, Fig 5). Its mean migration date is 9 December in the late 20^th^ century, ranges from 18 December in GFDL to 5 January in MIROC5 for the mid-21^st^ century, and ranges from 5 January in GFDL to never in MIROC5 for the late 21^st^ century. Overwintering in the Great Lakes region could become increasingly likely by the late 21^st^ century for both American Black Ducks and Mallards (Table 1, Figs 5 and S1). The models with the least warming, such as CNRM and MRI, generally produce more modest delays in migration than the models with the greatest warming, such as MIROC5. For example, by the late 21^st^ century, the mean migration date of the Northern Shoveler in the Great Lakes region may become delayed by 21 days according to CNRM or 37 days according to MIROC5.

## Discussion and Conclusions

Projected changes in the relative abundance and timing of autumn-winter migration for seven dabbling duck species are investigated for the mid- and late 21^st^ century across the Mississippi and Atlantic Flyways. Based on aerial and ground-based survey data of waterfowl abundance, species-specific empirical relationships are established, specific to the Mississippi and Atlantic Flyways, between the rate of change in abundance of dabbling ducks and cumulative weather severity indices, based on daily mean air temperature, the number of consecutive days with mean air temperature below freezing, daily snow depth, and the number of consecutive days with at least 2.54 cm of snow on the ground. These weather severity indices reflect both energy conservation theory and energy acquisition theory by capturing the impacts of cold atmospheric conditions on the energy expenditure of ducks and of snow cover, lake ice cover, and wetland icing on food availability to ducks [12]. Of the seven species, the Northern Shoveler is the earliest migrant, as it is a wetland-obligate foraging species, which consumes invertebrates on or near the surface in shallow wetlands and must migrate once wetlands freeze. In contrast, the American Black Duck and Mallard are the latest migrants, acting as opportunistic foragers that can switch to waste agricultural grains when wetland foods are restricted, thereby enabling a delay in their autumn migration [22,104–106].

Statistical downscaling can be a powerful and efficient way to translate GCM output to spatial scales that are more relevant for planners and resource managers. However, a key assumption of statistical downscaling is that the drivers of local spatial variation in climate remain unchanged over time. This stationarity assumption has not held true for the Great Lakes Basin, where lake ice cover has rapidly declined in recent decades, leading to enhanced evaporation and lake-effect snowfall [82–85]. Past spatial variations in snowfall across the basin were largely driven by lakes that were significantly ice covered during the winter, but that is not expected in the future. This was a weakness of the study by [81], which was rectified in a small initial pool of dynamically downscaled models presented by [86,87]. The clear advantage of the dynamical downscaling approach is that it addresses projected changes in Great Lakes’ water temperatures, ice cover, and lake evaporation and resulting impacts on lake-effect snowfall. Changes in snowpack, lake ice cover, and wetland icing in the Great Lakes Basin have critical implications to food availability for dabbling ducks within the Mississippi and Atlantic Flyways. Here, the output from six CMIP5 global climate models is dynamically downscaled over much of the contiguous United States and southern Canada for the late 20^th^, mid-21^st^, and late 21^st^ centuries using a high-resolution regional climate model, RegCM4, interactively coupled to a one-dimensional lake model to represent the Great Lakes. This effort expands upon the initial pool of downscaled models presented by [86,87].

Dramatic reductions in weather severity are projected for the mid- and late 21^st^ century across the Mississippi and Atlantic Flyways, including substantial mean warming during autumn-winter, fewer days below freezing, and diminished lake ice cover, lake-effect snowfall, and snow depth. The most pronounced reductions in the number of days with snow on the ground is identified around 44–45°N, across the Great Lakes region, consistent with the findings of [81]. As discussed by [81], this zone is located close to the -5°C isotherm of the late 20^th^ century climatological near-surface air temperature for December-March, such that as the isotherm shifts northward in the future, projected trends in declining snowfall accelerate in this zone. Further to the north, mean temperatures remain low enough, even later this century, for much of the wintertime precipitation to still occur as snow and for the snowpack to persist. From a climatological perspective, we recommend that future work expand the number of applied RCMs, to better capture the spread of climate change uncertainty for the study region, and couple the RCM to a three-dimensional lake model, in order to represent the circulation of deep lakes and reduce biases in lake temperatures, timing of stratification, and ice cover.

The projected reduction in weather severity should lead to delayed autumn-winter migration for all seven dabbling duck species, with results suggesting that the delays will be least for the Northern Pintail and greatest for the Mallard (greatest delays for the ducks with the largest mean weight, namely American Black Duck and Mallard). By the late 21^st^ century, overwintering in the Great Lakes region may become increasingly likely for the American Black Duck and Mallard. This appears to be supported by the study of [67], which noted observed mid-winter trends toward an increased abundance of American Black Ducks in Ontario and decreased abundance in the Mississippi Flyway of the United States. The earlier study by [12], which lacked consideration of future changes in snow depth, likewise concluded that the Mallard would experience a greater northward shift in distribution than most other dabbling duck species, which generally migrate south during milder conditions than the Mallard. Future declines in wetland icing will also provide greater foraging opportunities for all species, but may benefit wetland obligates, like Gadwall and Northern Shoveler, to a greater degree [21–22]. Projected reductions in weather severity and delays in autumn-winter duck migration will increase foraging pressures on remaining wetland habitats in the Great Lakes Basin and Upper Midwest United States, but reduce energy needs in southerly locations [12].

Large economic losses might be expected for the southern flyway states due to diminished hunter and bird watching opportunity, especially because the most common species of duck in North America is projected to overwinter in some years in the Great Lakes Basin. This could reduce the number of migrating waterfowl in more southerly states significantly. As emphasized by [107], in light of ongoing climate change and northward shifts in duck distributions, it will be critical to protect and restore wetlands across the mid-latitudes of North America, especially given the loss of historic wetlands and ongoing stresses from development and pollution to existing wetlands across the Great Lakes region [1,75–76,108].

## Supporting Information

S1 Fig Probability of a negative population rate of the American Black Duck across the Mississippi and Atlantic Flyway, within latitudinal bands from 30°N to 50°N, for the late 20^th^, mid-21^st^, and late 21^st^ centuries (first three columns). The probabilities are shown for each day from 1 September through 31 March, with the x-axis labeled as days since 1 September. Projected changes in these probabilities are shown for the mid-21^st^ and late 21^st^ century, compared to the late 20^th^ century (fourth and fifth columns). Results are shown for CNRM, MIROC5, IPSL, MRI, ACCESS, and GFDL, from top to bottom row.
(EPS)

S2 Fig Probability of a negative population rate of the American Wigeon across the Mississippi and Atlantic Flyway, within latitudinal bands from 30°N to 50°N, for the late 20^th^, mid-21^st^, and late 21^st^ centuries (first three columns). The probabilities are shown for each day from 1 September through 31 March, with the x-axis labeled as days since 1 September. Projected changes in these probabilities are shown for the mid-21^st^ and late 21^st^ century, compared to the late 20^th^ century (fourth and fifth columns). Results are shown for CNRM, MIROC5, IPSL, MRI, ACCESS, and GFDL, from top to bottom row.
(EPS)

S3 Fig Probability of a negative population rate of the Gadwall across the Mississippi and Atlantic Flyway, within latitudinal bands from 30°N to 50°N, for the late 20^th^, mid-21^st^, and late 21^st^ centuries (first three columns). The probabilities are shown for each day from 1 September through 31 March, with the x-axis labeled as days since 1 September. Projected changes in these probabilities are shown for the mid-21^st^ and late 21^st^ century, compared to the late 20^th^ century (fourth and fifth columns). Results are shown for CNRM, MIROC5, IPSL, MRI, ACCESS, and GFDL, from top to bottom row.
(EPS)

S4 Fig Probability of a negative population rate of the Green-Winged Teal across the Mississippi and Atlantic Flyway, within latitudinal bands from 30°N to 50°N, for the late 20^th^, mid-21^st^, and late 21^st^ centuries (first three columns). The probabilities are shown for each day from 1 September through 31 March, with the x-axis labeled as days since 1 September. Projected changes in these probabilities are shown for the mid-21^st^ and late 21^st^ century, compared to the late 20^th^ century (fourth and fifth columns). Results are shown for CNRM, MIROC5, IPSL, MRI, ACCESS, and GFDL, from top to bottom row.
(EPS)

S5 Fig Probability of a negative population rate of the Northern Pintail across the Mississippi and Atlantic Flyway, within latitudinal bands from 30°N to 50°N, for the late 20^th^, mid-21^st^, and late 21^st^ centuries (first three columns). The probabilities are shown for each day from 1 September through 31 March, with the x-axis labeled as days since 1 September. Projected changes in these probabilities are shown for the mid-21^st^ and late 21^st^ century, compared to the late 20^th^ century (fourth and fifth columns). Results are shown for CNRM, MIROC5, IPSL, MRI, ACCESS, and GFDL, from top to bottom row.
(EPS)

S1 File Netcdf file containing underlying data from Fig 2A–2C. (NC)

S2 File Netcdf file containing underlying data from Fig 2D–2F. (NC)

S3 File Excel file containing underlying data from Fig 3. (XLSX)

S4 File Netcdf file containing underlying data from Fig 4A–4C. (NC)

S5 File Netcdf file containing underlying data from Fig 4D–4F. (NC)

S6 File Netcdf file containing underlying data from Fig 5. (NC)

S7 File Netcdf file containing underlying data from Fig 6. (NC)

S1 Table Sample daily data for a location in Wisconsin (44.79°N, 89.95°W) to demonstrate the calculation of cumulative weather severity indices. Here, the data includes the day since 1 September, daily mean air temperature (°C), daily snow depth (cm), four components of weather severity (TEMP, TEMPDAY, SNOW, and SNOWDAY), the PC1 index, and computed population rate of change for Mallards. Grey shading indicates conditions favorable for declining populations related to migration. It might be expected that Mallards would migrate south from this location around early December that year.
(DOCX)
