# Supplementary material for: Projected Influences of Changes in Weather Severity on Autumn-Winter Distributions of Dabbling Ducks in the Mississippi and Atlantic Flyways during the Twenty-First Century
Source: PLoS One. 2016 Dec 13;11(12):e0167506. doi: 10.1371/journal.pone.0167506 (PMC5154525; doi:10.1371/journal.pone.0167506)
Supplement: S1 Table — Here, the data includes the day since 1 September, daily mean air temperature (°C), daily snow depth (cm), four components of weather severity (TEMP, TEMPDAY, SNOW, and SNOWDAY), the PC1 index, and computed population rate of change for Mallards. Grey shading indicates conditions favorable for declining populations related to migration. It might be expected that Mallards would migrate south from this location around early December that year. (DOCX) [file pone.0167506.s013.docx]

**S1 Table.** **Sample daily data for a location in Wisconsin (44.79°N, 89.95°W) to demonstrate the calculation of cumulative weather severity indices.**

| **Day Since 1 Sep** | **Air T (°C)** | **Snow Depth (cm)** | **TEMP** | **TEMPDAY** | **SNOW** | **SNOWDAY** | **PC1** | **Population Rate** |
| --- | --- | --- | --- | --- | --- | --- | --- | --- |
| 68 | 1.15 | 0.00 | -1.15 | 0 | 0.00 | 0 | -1.08 | 1.04 |
| 69 | -2.06 | 0.00 | 2.06 | 1 | 0.00 | 0 | 2.16 | 0.85 |
| 70 | 1.51 | 0.28 | -1.51 | 0 | 0.11 | 0 | -1.40 | 1.05 |
| 71 | -4.18 | 0.19 | 4.18 | 1 | 0.08 | 0 | 4.13 | 0.64 |
| 72 | -1.02 | 0.20 | 1.02 | 2 | 0.08 | 0 | 1.43 | 0.90 |
| 73 | 2.95 | 0.20 | -2.95 | 0 | 0.08 | 0 | -2.74 | 1.08 |
| 74 | 2.06 | 0.00 | -2.06 | 0 | 0.00 | 0 | -1.92 | 1.06 |
| 75 | 0.83 | 0.00 | -0.83 | 0 | 0.00 | 0 | -0.77 | 1.03 |
| 76 | 1.71 | 0.00 | -1.71 | 0 | 0.00 | 0 | -1.59 | 1.06 |
| 77 | 1.55 | 0.00 | -1.55 | 0 | 0.00 | 0 | -1.45 | 1.05 |
| 78 | -0.85 | 0.00 | 0.85 | 1 | 0.00 | 0 | 1.02 | 0.93 |
| 79 | -3.61 | 0.00 | 3.61 | 2 | 0.00 | 0 | 3.83 | 0.68 |
| 80 | -7.00 | 0.00 | 7.00 | 3 | 0.00 | 0 | 7.23 | 0.20 |
| 81 | -3.46 | 0.06 | 3.46 | 4 | 0.02 | 0 | 4.16 | 0.64 |
| 82 | 0.07 | 0.48 | -0.07 | 0 | 0.19 | 0 | -0.06 | 1.00 |
| 83 | 0.42 | 0.62 | -0.42 | 0 | 0.24 | 0 | -0.38 | 1.01 |
| 84 | -1.16 | 0.64 | 1.16 | 1 | 0.25 | 0 | 1.33 | 0.91 |
| 85 | -0.04 | 1.00 | 0.04 | 2 | 0.39 | 0 | 0.53 | 0.97 |
| 86 | -12.84 | 1.12 | 12.84 | 3 | 0.44 | 0 | 12.70 | -0.95 |
| 87 | -12.40 | 1.16 | 12.40 | 4 | 0.46 | 0 | 12.52 | -0.91 |
| 88 | -6.07 | 1.30 | 6.07 | 5 | 0.51 | 0 | 6.86 | 0.26 |
| 89 | -3.60 | 1.38 | 3.60 | 6 | 0.54 | 0 | 4.80 | 0.56 |
| 90 | -4.96 | 1.43 | 4.96 | 7 | 0.56 | 0 | 6.30 | 0.35 |
| 91 | -4.41 | 1.51 | 4.41 | 8 | 0.59 | 0 | 6.03 | 0.39 |
| 92 | -5.60 | 2.06 | 5.60 | 9 | 0.81 | 0 | 7.38 | 0.18 |
| 93 | -16.92 | 2.51 | 16.92 | 10 | 0.99 | 0 | 18.17 | -2.59 |
| 94 | -21.39 | 2.50 | 21.39 | 11 | 0.98 | 0 | 22.58 | -4.25 |
| 95 | -14.57 | 2.52 | 14.57 | 12 | 0.99 | 0 | 16.45 | -2.02 |
| 96 | -13.42 | 2.55 | 13.42 | 13 | 1.00 | 1 | 15.89 | -1.85 |
| 97 | -9.74 | 2.54 | 9.74 | 14 | 1.00 | 2 | 12.97 | -1.02 |
| 98 | -16.20 | 2.66 | 16.20 | 15 | 1.05 | 3 | 19.50 | -3.05 |
| 99 | -21.67 | 2.53 | 21.67 | 16 | 1.00 | 0 | 24.02 | -4.86 |
| 100 | -24.08 | 2.37 | 24.08 | 17 | 0.93 | 0 | 26.49 | -5.99 |
| 101 | -13.05 | 2.20 | 13.05 | 18 | 0.87 | 0 | 16.45 | -2.02 |
| 102 | -4.00 | 2.09 | 4.00 | 19 | 0.82 | 0 | 8.25 | 0.02 |
| 103 | -9.40 | 2.07 | 9.40 | 20 | 0.81 | 0 | 13.52 | -1.17 |
| 104 | -12.11 | 3.11 | 12.11 | 21 | 1.23 | 1 | 16.57 | -2.06 |
| 105 | -17.28 | 3.00 | 17.28 | 22 | 1.18 | 2 | 21.89 | -3.97 |
| 106 | -23.73 | 2.92 | 23.73 | 23 | 1.15 | 3 | 28.41 | -6.93 |
| 107 | -29.05 | 2.83 | 29.05 | 24 | 1.12 | 4 | 33.87 | -9.93 |
| 108 | -13.70 | 3.35 | 13.70 | 25 | 1.32 | 5 | 20.08 | -3.27 |
| 109 | -3.49 | 3.47 | 3.49 | 26 | 1.36 | 6 | 11.06 | -0.56 |
| 110 | -1.68 | 3.47 | 1.68 | 27 | 1.37 | 7 | 9.88 | -0.30 |
| 111 | -0.83 | 4.03 | 0.83 | 28 | 1.59 | 8 | 9.61 | -0.24 |
| 112 | -18.40 | 5.83 | 18.40 | 29 | 2.30 | 9 | 26.53 | -6.01 |
| 113 | -27.30 | 5.87 | 27.30 | 30 | 2.31 | 10 | 35.34 | -10.82 |
| 114 | -13.98 | 6.10 | 13.98 | 31 | 2.40 | 11 | 23.42 | -4.60 |
| 115 | -19.88 | 7.54 | 19.88 | 32 | 2.97 | 12 | 29.46 | -7.47 |
| 116 | -21.16 | 7.77 | 21.16 | 33 | 3.06 | 13 | 31.16 | -8.38 |
| 117 | -4.93 | 9.51 | 4.93 | 34 | 3.74 | 14 | 16.58 | -2.06 |
| 118 | -0.91 | 8.36 | 0.91 | 35 | 3.29 | 15 | 13.31 | -1.11 |
| 119 | -0.18 | 7.03 | 0.18 | 36 | 2.77 | 16 | 13.11 | -1.06 |
| 120 | -6.71 | 7.61 | 6.71 | 37 | 3.00 | 17 | 19.71 | -3.13 |
| 121 | -6.53 | 8.65 | 6.53 | 38 | 3.41 | 18 | 20.07 | -3.27 |
| 122 | -1.87 | 8.17 | 1.87 | 39 | 3.22 | 19 | 16.23 | -1.95 |
| 123 | -8.09 | 7.18 | 8.09 | 40 | 2.83 | 20 | 22.51 | -4.22 |
| 124 | -14.13 | 6.85 | 14.13 | 41 | 2.70 | 21 | 28.64 | -7.05 |
| 125 | -3.48 | 6.93 | 3.48 | 42 | 2.73 | 22 | 19.22 | -2.95 |
| 126 | -15.86 | 6.52 | 15.86 | 43 | 2.57 | 23 | 31.25 | -8.43 |
| 127 | -6.41 | 6.29 | 6.41 | 44 | 2.48 | 24 | 22.95 | -4.40 |
| 128 | -11.89 | 6.23 | 11.89 | 45 | 2.45 | 25 | 28.55 | -7.00 |
| 129 | -13.81 | 6.06 | 13.81 | 46 | 2.39 | 26 | 30.85 | -8.22 |
| 130 | -8.49 | 6.01 | 8.49 | 47 | 2.36 | 27 | 26.39 | -5.94 |
| 131 | -7.94 | 6.01 | 7.94 | 48 | 2.37 | 28 | 26.39 | -5.94 |
| 132 | -12.19 | 5.95 | 12.19 | 49 | 2.34 | 29 | 30.86 | -8.22 |
| 133 | -8.06 | 5.99 | 8.06 | 50 | 2.36 | 30 | 27.51 | -6.48 |
| 134 | -6.87 | 6.63 | 6.87 | 51 | 2.61 | 31 | 26.92 | -6.20 |
| 135 | -16.47 | 7.44 | 16.47 | 52 | 2.93 | 32 | 36.39 | -11.48 |
| 136 | -26.33 | 7.51 | 26.33 | 53 | 2.96 | 33 | 46.09 | -18.38 |
| 137 | -26.70 | 7.56 | 26.70 | 54 | 2.98 | 34 | 46.94 | -19.06 |
| 138 | -12.57 | 7.84 | 12.57 | 55 | 3.09 | 35 | 34.28 | -10.18 |
| 139 | -4.01 | 8.06 | 4.01 | 56 | 3.17 | 36 | 26.81 | -6.14 |
| 140 | -6.23 | 8.66 | 6.23 | 57 | 3.41 | 37 | 29.40 | -7.44 |
| 141 | -15.10 | 8.70 | 15.10 | 58 | 3.42 | 38 | 38.17 | -12.64 |
| 142 | -16.39 | 9.10 | 16.39 | 59 | 3.58 | 39 | 39.89 | -13.80 |
| 143 | -13.86 | 11.39 | 13.86 | 60 | 4.49 | 40 | 38.09 | -12.58 |
| 144 | -13.50 | 11.62 | 13.50 | 61 | 4.58 | 41 | 38.26 | -12.69 |
| 145 | -15.98 | 11.63 | 15.98 | 62 | 4.58 | 42 | 41.08 | -14.63 |
| 146 | -17.13 | 11.64 | 17.13 | 63 | 4.58 | 43 | 42.65 | -15.77 |
| 147 | -24.23 | 11.63 | 24.23 | 64 | 4.58 | 44 | 49.78 | -21.40 |
| 148 | -27.61 | 11.59 | 27.61 | 65 | 4.56 | 45 | 53.44 | -24.61 |
| 149 | -19.52 | 13.04 | 19.52 | 66 | 5.13 | 46 | 46.43 | -18.65 |

Here, the data includes the day since 1 September, daily mean air temperature (°C), daily snow depth (cm), four components of weather severity (TEMP, TEMPDAY, SNOW, and SNOWDAY), the PC1 index, and computed population rate of change for Mallards. Grey shading indicates conditions favorable for declining populations related to migration. It might be expected that Mallards would migrate south from this location around early December that year.
